# Metagenomics in ophthalmology: current findings and future prospectives

**DOI:** 10.1136/bmjophth-2018-000248

**Published:** 2019-06-04

**Authors:** Davide Borroni, Vito Romano, Stephen B Kaye, Tobi Somerville, Luca Napoli, Adriano Fasolo, Paola Gallon, Diego Ponzin, Alfonso Esposito, Stefano Ferrari

**Affiliations:** 1 St Paul's Eye Unit, Department of Corneal and External Eye Diseases, Royal Liverpool University Hospital, Liverpool, United Kingdom; 2 Department of Doctoral Studies, Riga Stradins University, Riga, Latvia; 3 Department of Eye and Vision Science, University of Liverpool, Liverpool, United Kingdom; 4 Fondazione Banca Degli Occhi Del Veneto Onlus, Zelarino, Venezia, Italy; 5 Dipartimento di Specialità Medico-Chirurgiche, Scienze Radiologiche e Sanita Pubblica, Universita degli Studi di Brescia, Brescia, Italy; 6 Centre for Integrative Biology (CIBIO), Trento University, Trento, Italy

**Keywords:** ophthalmology, metagenomics, sequencing, eye, ocular surface, cornea, microbiome

## Abstract

Less than 1% of all microorganisms of the available environmental microbiota can be cultured with the currently available techniques. Metagenomics is a new methodology of high-throughput DNA sequencing, able to provide taxonomic and functional profiles of microbial communities without the necessity to culture microbes in the laboratory. Metagenomics opens to a ‘hypothesis-free’ approach, giving important details for future research and treatment of ocular diseases in ophthalmology, such as ocular infection and ocular surface diseases.

## Current knowledge about the eye microbiome

The ocular surface (OS) microbiome is an understudied topic, compared with other host-associated environments. While the *Human Microbiome Project* initially studied five main body areas—the skin, the gastrointestinal tract, the urogenital tract, the oral and the nasal mucosa^1^—an emerging area of research is focusing on the eye and the microbiota of the OS.^2^

**Figure 1:**
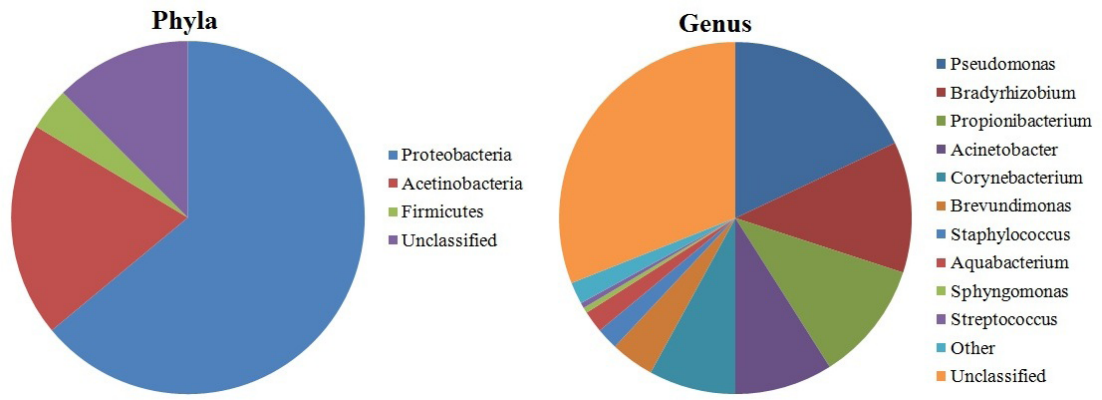
Pie charts displaying the relative proportions of taxa at the phylum level and at the genus level in healthy eye microbiome (according to Dong *et al* 15 [15]).

Recent studies demonstrated that OS hosts a number of commensal microorganisms.^3^ Earlier culture-based surveys suggested that the OS are colonised by microbial communities dominated by Gram-positive Firmicutes, in particular, species belonging to the *Staphylococcus, Streptococcus*, *Corynebacterium* and *Propionibacterium*.^4^ A screening including approximately 1000 16S rRNA reads revealed that the diversity of healthy conjunctiva was higher than previously thought.^5^ Other recent studies based on traditional microbiological techniques have examined the microbiota of the OS,^6–11^ although a more comprehensive analysis of microbial diversity of OS has been hindered by the limitations of conventional cultivation techniques.^12–14^ More recent screening of OS-associated microbiome, using molecular metagenomic techniques, extended further the knowledge about OS microbial diversity.^2 5 15 16^ Shestopalov and colleagues estimated using real-time PCR that in 1 ng of extracted DNA, the number of bacterial genomes (ie, bacterial richness) was on average 79.8 and 729 in the conjunctiva and cornea, respectively. Significant amounts (22 over 55) were detected in the eye for the first time.^17^ Dong *et al* detected 59 distinct bacterial genera using a 16S rDNA gene pyrosequencing approach on the OS of four healthy individuals^15^ (figure 1). Despite the low number of individuals examined, this is one of the first studies focusing on the bacterial diversity of the OS microbiome. Healthy OS microbiome is dominated by *Proteobacteria*, *Actinobacteria* and *Firmicutes*. The most common taxa at the genus level were *P seudomonas, Propionibacterium, Bradyrhizobium, Corynebacterium, Acinetobacter, Brevundimonas, Staphylococci, Aquabacterium, Sphingomonas, Streptococcus, Streptophyta* and *Methylobacterium* (figure 1). This is in general agreement with the previous studies, although many false positives may derive from contamination.^4^

Microbial invasion into the OS compromises corneal clarity and causes inflammation in blinding conditions like keratitis, endophthalmitis and trachoma. Herpes simplex type 1, hepatitis B and C viruses can be detected using PCR in the tears of asymptomatic volunteers,^18^ thus suggesting that simply focusing solely on the bacterial constituents of the OS may result in an incomplete understanding of the OS microbiome. In recent time, Zhou *et al* 19 showed how the changes in the conjunctival microbiome occur in trachomatous disease compared with normal controls. Wen *et al* 20 showed how the microbiome of healthy OS is shaped by age and sex and how the ocular microbiome of house finches changed during experimentally induced mycoplasmal conjunctivitis.^21^

Study of microbiome in OS disease has significant potential to improve the diagnosis, treatment and management of potential blinding diseases.

## Background and evolution of metagenomics

The non-culturability of microbes dates back to 1898 by Heinrich Winterberg, formalised as the ‘*great plate count anomaly’* by Staley and Konopka in 1985.^22^ Several theories have been proposed to explain the non-culturability of microbes: (1) the cultural media used have been developed to grow microorganisms usually involved in human or animal diseases, missing a diversity of other microorganisms; (2) some bacteria may require long incubation time to form visible colonies; (3) physiological features (ie, obligate symbiosis with other species or strong quorum sensing signalling processes) may hinder the cultivation of some species; and (4) some bacteria may require a specific combination of nutritional features and aerobic requirements. The pioneering work by Woese^23^ in 1985 identified the 16S rRNA gene as an evolutionary chronometer for bacterial phylogeny. This gene has three unique features: (A) the ubiquity in the bacterial kingdom, (B) the structure of the gene itself, made of both variable and conserved regions (this is due to the secondary structure of the transcribed RNA, made of stretches and loops), and (C) the low (if any) amount of horizontal gene transfer. Pace *et al* in 1985 had the idea that 16S rRNA gene isolated from the environmental samples can directly be cloned.^24^ In 1991, Schmidt *et al* 25 successfully cloned 16S rRNA gene sequences from marine picoplankton with the use of bacteriophage lambda vector.^26^ In 1998, the term metagenome was introduced.^27^ In the last two decades metagenomic analyses have been performed on majority of the natural environments, for example, soils,^28^ marine picoplankton,^29^ hot springs,^30^ surface water from rivers,^31^ glacier ice,^32^ Antarctic desert soil^33^ and gut of ruminants.^26 34^ Earlier, major parts of the metagenomic studies were based on low-throughput approaches, like terminal restriction fragment-length polymorphism analysis,^35^ denaturing gradient gel electrophoresis^36^ or Sanger sequencing.^37^ The comparison with the sequences included in curated databases like the ones of Ribosomal Database Project II,^38^ Greengenes^39^ and SILVA^40^ has allowed the taxonomic classification and community profiling of environmental 16S rRNA gene sequences.

## Clinical metagenomics and its potential as diagnostic tool

Classical microbiological methods are able to identify only the presumed cause of ocular infection in about 40% cases. In contrast, a metagenomic approach promises to provide important detail regarding all microbiota, allowing the identification of a greater portion of previously unidentified and the so-called ‘uncultured majority’ of microorganisms,^41^ whether being prokaryotes, eukaryotes or viruses. Efficient next-generation sequencing (NGS) technologies have developed greatly in the last decade, along with a reduced cost, gaining interest in the scientific community in several fields (such as medicine, biotechnology, agriculture or genetics).^42^ The deep impact on metagenomics given by the NGS technologies allowed the study of microbes with a much higher throughput. This enabled a ‘hypothesis-free’ approach providing all the components of the microbiome. The data produced by NGS have also proved to be particularly suitable for taxonomic and functional profiling. Thus, metagenomics opened the way to explore the remarkable genetic potential of bacteria; in some cases it was used to get hints on the metabolic requirements of yet-to-be cultured bacteria.^43^ More often, the application of NGS to metagenomics studies aimed to profile and compare taxonomy and function of microbial communities from different sources.^23^ For example, comparing human samples from a disease state to samples from healthy controls allows clinicians to get a more holistic view of the quantitative shifts of specific taxa during the course of the disease.^1 44^ The extraction of DNA from a human-derived sample is usually the first step of the workflow, then two possible downstream analyses can be done: (1) amplify a marker gene (usually a portion of the rRNA genes) and sequence the PCR amplification product; or (2) sequencing the extracted, fragmented DNA directly (this approach is known as ‘shotgun’ metagenomics sequencing).

There is a wealth of studies on the human microbiome, spanning several physiological conditions related to age, disease, race and many more variables. Starting from the first ‘inocula’, that is, transmission from mothers to children.^45^ A significant role has been attributed to the birth delivery method, with marked differences in microbiota between children born through vaginal delivery versus caesarean section.^46^ Different studies have debated determining the ‘core’ bacterial taxa of gut and skin.^1 47^ The latest analysis suggests that instead of the core taxa, homeostatic communities are defined by the presence of a core microbial gene set that encodes essential metabolic pathways.^12 48^

In addition, several studies have highlighted the variability of the microbiome according to body sites, race and ethnicity as outlined by Gupta *et al*.^49^ The genetic asset of the host itself influences the microbiome; this is well studied in metabolic dysfunctions such as obesity, diabetes and inflammatory bowel disease.^50^ Two simultaneous projects: the European project, MetaHIT (Metagenomics of the Human Intestinal Tract—www.metahit.eu), and the American Human Microbiome Project,^51^ use metagenomics to facilitate the study of human intestinal microbiome. The use of drugs has also an influence on the microbiome. Bioinformatic tools are being developed and updated almost monthly for the analysis of the data^52 53^ and in developing integrated databases, for example, https://portal.hmpdacc.org/. They are, however, over-represented by strong biases towards samples from stool, and oral and vaginal microbiomes.

## Selecting the test

The two methods (shotgun and marker-based metagenomics) can be used in different instances: the marker-based approach is used to get the taxonomic profiles of the community under study, whereas shotgun approach gives wider information on function and an extended phylogenetic breadth.^54^ For both methods, there are pros and cons: marker-based studies are well suited for analysis of large number of samples, that is, multiple patients, longitudinal studies, and so on, and are cheaper; however, there are well-known amplification biases and the amount of information is limited to the taxonomy.^55^ On the other hand, shotgun metagenomics is usually more expensive. It may miss low-abundant species and when host-associated metagenomes are studied, most of the reads derive from the host genome, especially when studying sites with low bacterial biomass. It offers, however, increased resolution, enabling the possibility to discover new microbial genes and genomes as well as a more specific taxonomic and functional classification of sequences (in some cases). Importantly, shotgun metagenomics allows the simultaneous study of viruses, bacteriophages, archaea and eukaryotes.^56^ Sample collection and storage methods are critical for most metagenomic studies: they are often arbitrary and rely on the common practices developed in single laboratories or even by single researchers.^57^ However, in some cases, such as the study of the human faecal microbiome, there are well-established standard procedures.^58^

A standardised protocol for sample collection, handling and storage for metagenomic studies in ophthalmology is still under development (data not shown). In addition, as all low biomass samples, corneal surfaces are particularly vulnerable to external contaminations, which could also derive from the reagent kits,^59^ therefore, a proper experimental design should include a number of blank controls and the use of ultrapure reagents to minimise this risk. Several significant efforts to unravel bacterial identity with a resolution as high as the level of strain have already been published.^60^ The integration of the metaomics (collective name that stands for metagenomics, metatranscriptomics, metaproteomics, and so on) with information such as clinical history, dietary information and genetic background of the patient may be useful in the implementation of mechanistic models explaining the microbiome structure and function.^61^ Biomarker discovery needs a high number of replicates; one pipeline developed for this task is LEfSe^62^ which relies on the linear discriminant analysis of effect size. It detects consistent abundance patterns among features (that can be either taxa or coding genes) in a multidimensional data set such as a species-per-sample metagenomic matrix. It is highly scalable and it has proved to achieve a discrete performance in reducing the false-positive detection, although as explicitly admitted by the developer, the false-negative rate is slightly higher. Other pipelines are also available for biomarker discoveries,^63^ however, a benchmark among them is beyond the scope of this review. Last, but not least, the complex tasks described above require high computational power and specific expertise in the field of biostatistics and informatics.^64^

## From bench to bedside: clinical applications

### Clinical applications in OS

Doan and Pinsky in 2016 showed how the healthy OS has a unique microbiome with viral and bacterial communities. In their study, quantitative 16S PCR resulted in 0.1 bacterial 16S rDNA/human actin copy on the OS compared with 10 16S rDNA/human actin copies of facial skin and higher bacterial diversity on the OS.^65^ Many ocular infections are acquired on the OS and the diagnosis of causative pathogen can be challenging.^66 67^ In postoperative endophthalmitis, the pathogen identification is between 50% and 70%^68 69^ and cultures failed in approximately 33% of cases.^70^ In a recent study on patients with uveitis, Doan *et al* found that herpes simplex virus type 1 (HSV-1), *Cryptococcus neoformans* and *Toxoplasma gondii* were associated with the disease.^71^ Other than Rubeola RNA virus, even Ebola RNA virus was detected in the ocular fluid after resolution of viraemia.^72^ A recent attempt provided a proof of concept for the use of metagenomics as diagnostic tool, and developed specific bioinformatic pipelines to differentiate pathogenic agents and antibiotic resistance genes with a higher resolution.^73^

### Contact lens

Shin *et al* showed that wearing contact lenses makes ocular conjunctiva more similar to the skin microbiota.^16^ Lee *et al* 74 studied blepharitis using different sampling from eyelashes and tears showing increased *Staphylococcus*, *Streptophyta*, *Corynebacterium* and *Enhydrobacter*. Studies suggested how contact lenses could function as a medium for skin bacteria to come to OS.^75 76^ Zhang *et al* found how slight microbe variability was found between orthokeratology lens wearers with soft contact lenses wearers and in non-wearers.^77^

## Connection between gut and eye microbiome

It is well acknowledged that gut microbiome influences the communication between the enteric nervous system and the central nervous system (known as gut-brain axis).^78^ Likewise, OS microbiome shows connection to the gut microbiome. Considering that the eye is the site of inflammatory diseases like uveitis, scleritis and Mooren’s corneal ulcer, it is possible that these autoimmune reactions are associated with dysbiosis in the gut.^79^ de Paiva *et al* found that ‘*the severity of Sjögren Syndrome (SS) ocular and systemic disease was inversely correlated with microbial diversity.*’^80^ SS is marked by a dysbiotic intestinal microbiome driven by low relative abundance of commensal bacteria and high relative abundance of potential pathogenic genera. This is associated with worse ocular mucosal disease in a mouse model of SS and in patients with SS. The lowest diversity of stool microbiota was found in subjects with the most severe keratoconjunctivitis sicca and combined systemic and ocular disease. Such result is in agreement with other findings in which a disease state correlates with the low diversity of the microbiome in a specific compartment, such as inflammatory bowel disease (where *Clostridium difficile* dominates the microbiome) or the pulmonary microbiome during cystic fibrosis.^81^ Animal models of experimental autoimmune uveitis have shown significant attenuation of this disease following administration of oral antibiotics that altered the intestinal microbiota.^82^ Further evidence strongly suggests that the homeostatic microbiome plays a protective role in preventing colonisation of pathogenic species. It was demonstrated that oral administration of antibiotics reduced the severity of uveitis in mice with experimentally induced autoimmune uveitis.^83 84^ The recent cases of persistent infection with Ebola virus,^72^ and possibly Zika virus,^85^ explain the urgency to develop better diagnostics for uveitis. These cases, with important public health consequences, highlight the eye’s role as a potential reservoir for infectious agents. In addition, epigenetic mechanisms may cooperate with microbiota to initiate ocular inflammation.^86^

## Discussions and conclusion

Detection of intraocular infections relies heavily on molecular diagnostics. In ophthalmology, the most widely available molecular diagnostic panel to detect infections includes separate pathogen-directed PCRs: HSV, varicella zoster virus, cytomegalovirus and *T*. *gondii*. Not surprisingly, *more than 50% of all presumed corneal infections fail to have a pathogen isolated*.^87 88^ NGS has offered clear advantages to make a definite diagnosis compared with conventional diagnostic methods and advances in metagenomics have made the use of NGS more useful for clinical disgnostics.^71^ Metagenomic deep sequencing (MDS) has the potential to improve diagnostic yield; it can theoretically detect all pathogens in a clinical sample^89^ using an unbiased and hypothesis-free approach. Wilson *et al* showed that NGS protocols could be completed in less than 48 hours^90^ with a significant advantage compared with many of the culture-dependent assays. Improving our understanding of the composition and function of a normal ocular microbiome would be a good starting point for a targeted therapy and the development of probiotic products. Host-microbe and microbe-microbe interactions on the OS indicate the beneficial function of the microbiota and the understanding of these principles by the clinicians could possibly guide appropriate use of topical and systemic antibiotics.^91^

MDS could be used in patients with difficult-to-diagnose infections, as a front-line diagnostic tool. In difficult-to-culture samples, metagenomic shotgun is a promising test for the identification of microbial keratitis and undiagnosed encephalitis.^92 93^ Together with uveitis-related disorders, the microbiome study can potentially and dramatically change the management and treatment of these diseases.

**Figure 2:**
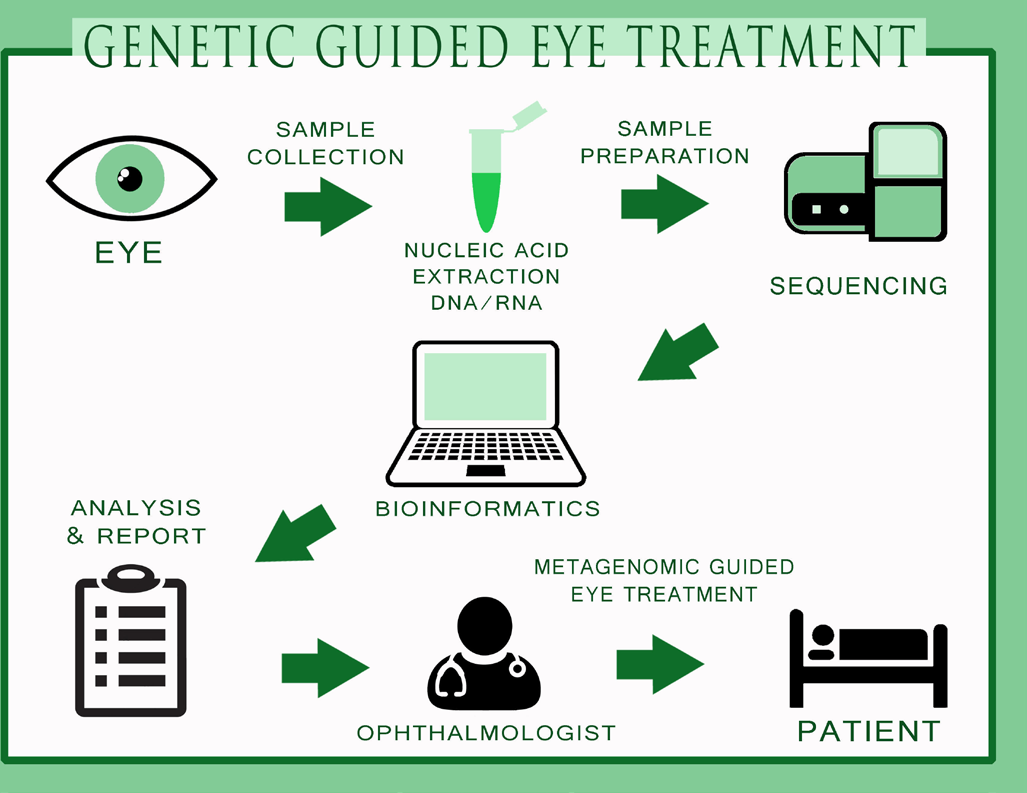
The process towards genetic-guided treatment in the field of ophthalmology. The figure indicates different procedures of sample collection, nucleic acid extraction, sample preparation, sequencing, bioinformatics, analysis and report writing, indication to the eye surgeon for specific drug usage for specific microorganism and metagenomic-guided eye treatment on the patient. Being highly specific and cost-effective, metagenomics could be potentially used in ophthalmology in the near future.

MDS may supplement or replace numerous and expensive diagnostic assays and procedures currently employed and improve patient outcomes. This approach will allow a far more comprehensive characterisation of the aetiology of infections and also complement the current diagnostic paradigm in ophthalmology. In the near future, a full genetic approach to eye infections is not far away (figure 2) where the samples will be collected, sequenced for specific targets and a specific antimicrobial used to treat the disease. With a certain clinical impact, the ophthalmologists will have precise quantification, multilocus sequence typing of single species and genetic sequence of the microbiota of ocular samples without the problems associated with tests based on conventional cultures. Hence, we foresee that metagenomics could further advance the field of ophthalmology especially in diagnosis and target-specific treatments.
