# Correction: Metagenomics in ophthalmology: current findings and future prospectives

**DOI:** 10.1136/bmjophth-2018-000248corr1

**Published:** 2019-06-27

Borroni D, Romano V, Kaye SB, *et al*. Metagenomics in ophthalmology: current findings and future prospectives. *BMJ Open Ophth* 2019;4:e000248. doi: 10.1136/bmjophth-2018-000248.

The license type for this paper has changed from CC BY-NC to CC BY.

